# Quantitative evaluation of *E. coli* F4 and *Salmonella* Typhimurium binding capacity of yeast derivatives

**DOI:** 10.1186/2191-0855-3-62

**Published:** 2013-10-22

**Authors:** Anja Ganner, Christian Stoiber, Jakob Tizian Uhlik, Ilse Dohnal, Gerd Schatzmayr

**Affiliations:** 1 BIOMIN Research Center, Technopark 1, 3430, Tulln, Austria

**Keywords:** Yeast cell wall, *E. coli* F4, *Salmonella* Typhimurium, Microplate-based assay

## Abstract

The target of the present study was to quantify the capacity of different commercially available yeast derivatives to bind *E. coli* F4 and *Salmonella* Typhimurium. In addition, a correlation analysis was performed for the obtained binding numbers and the mannan-, glucan- and protein contents of the products, respectively. In a subsequent experiment, different yeast strains were fermented and treated by autolysis or French press to obtain a concentrated yeast cell wall. The capacity of yeast cell wall products to bind *E. coli* F4 and *Salmonella* Typhimurium was assessed with a quantitative microbiological microplate-based assay by measuring the optical density (OD) as the growth parameter of adhering bacteria. Total mannan and glucan were determined by HPLC using an isocratic method and a Refractive Index (RI) Detector. Total protein was determined by Total Kjeldahl Nitrogen (TKN). Statistical analyses were performed with IBM SPSS V19 using Spearman correlation and Mann Whitney *U* Test.

Different yeast derivatives show different binding numbers, which indicate differences in product quality.

Interestingly, the binding numbers for *Salmonella* Typhimurium are consistently higher (between one and two orders of magnitude) than for *E. coli* F4.

We could demonstrate some statistical significant correlations between the mannan- and glucan content of different yeast derivatives and pathogen binding numbers; however, for the different yeast strains fermented under standardized laboratory conditions, no statistically significant correlations between the mannan- and glucan content and the binding numbers for *E. coli* and *Salmonella* Typhimurium were found.

Interestingly, we could demonstrate that the yeast autolysis had a statistically significant difference on *E. coli* binding in contrast to the French press treatment. *Salmonella* binding was independent of these two treatments.

As such, we could not give a clear statement about the binding factors involved. We propose that many more factors apart from mannan- and glucan content, such as cell wall structure, strain diversity, structural diversity, structural surroundings, and non-specific interactions play important roles in pathogen immobilization.

## Introduction

Yeast derivatives such as yeast cell wall products and recently also yeast autolysate products are sold worldwide as feed supplements, some of which claim to bind enteropathogenic bacteria such as *E. coli* and *Salmonella* spp.. Although yeast cell wall products have been in the market for a long time, their binding capacity and the mechanism of pathogen binding is still not well understood. There is much confusion throughout the animal feed industry concerning the quality of yeast products. An important initial event in bacterial pathogenesis is the adherence of bacteria via their surface lectins to host intestinal cells. Infections are initiated only after the microorganism has first adhered to the host cell surface. If this initial adherence can be inhibited, so can the subsequent infection. This approach forms the basis of anti-adherence strategies with the most studied being receptor-analogs, which include oligosaccharides (Shoaf-Sweeney and Hutkins 2008).

According to the literature, mannan-oligosaccharides (MOS) are the substances in the yeast cell wall responsible for the binding of pathogens via mannose specific type-I fimbriae (e.g. Snellings et al. 1997; Sharon and Ofek 2000). However, questions have been raised of late as to whether the mannan quantity in the yeast cell wall is responsible for immobilizing enteric pathogens. A point made so far was the lack of quantitative analytical methods to determine the total amount of bacteria bound by the yeast cell wall (Applegate et al*.*^2010^). Some qualitative test procedures such as the agglutination method are available to investigate the binding capacity of yeast products (e.g. Mirelman et al. 1980; Eshdat et al*.*^1981^; Oyofo et al. 1989; Spring et al. 2000; Peng et al. 2001). The agglutination method mixes bacteria with a cell wall suspension followed by visual evaluation of binding. Critical issue with this procedure is the poor solubility of the yeast cell wall in aqueous dilutions. Furthermore visual evaluation of binding is highly subjective because it is not possible to determine whether the bacteria are actually bound or whether they are merely in proximity to the yeast. Thus reproducibility is not given. It is necessary to obtain the quantitative measurement of bacterial adhesion in order to evaluate the binding capacity and quality of yeast derivatives. The quantitative *in vitro* assay developed by Ganner et al. (2010) was used in the present study to investigate different yeast cell wall products (MOS) and yeast autolysates for their capacity to bind *E. coli* F4 and *Salmonella* Typhimurium. This method is based on a microtiter plate, the insolubility of the cell wall material, with its high molecular weight, can be used to coat the wells. The wells are allowed to be incubated with the test bacteria, unbound bacteria are rinsed out, and the growth rate of bound bacteria is subsequently quantified by optical density reading. The accuracy and reproducibility of binding is given by automated evaluation of optical density. However, in vitro assays may be criticized by product end users as not accurately reflecting in vivo responses. This may be true in some cases, but in others it may partially be a reflection of assay bias attributable to media selection, duration, or substrate concentration. Another limitation of this assay is that the binding mechanisms involved cannot be elucidated, for this purpose, studies on a molecular level and detailed characterization of cell wall components have to be examined in future research studies.

*E. coli* F4 and *Salmonella* Typhimurium were chosen for the present study as they are important pathogens in the animal sciences and are known to cause severe economic losses in livestock production. Furthermore, the mannan-, glucan- and protein content of the yeast products were determined and statistical evaluation for correlation was performed with IBM SPSS v19.

To collect more information on binding characteristics, a separate experiment was performed in addition to evaluating mannan- and glucan- content; eight different yeast strains were fermented in the laboratory, and subjected to autolysis and French press treatment, to investigate the potential influence of yeast strains and production methods.

## Material and methods

### Yeast derivatives

Different types of commercially available yeast cell wall and autolysed yeast products were used. Due to a confidentiality agreement, the trade names and manufacturers have been omitted.

### Autolysis and French press of cell wall fragments

Cell wall fragments were produced in the laboratory scale under standard conditions from the following yeast strains: *Saccharomyces cerevisiae* Bio 19 (DSMZ 1848), *Klyveromyces marxianus* Bio 21 (DSMZ 5420), *Saccharomyces boulardii* CAN 214, *Saccharomyces boulardii* HA 282, *Saccharomyces boulardii* HA 283, *Trichosporon mycotoxinivorans* Bio 328 (DSMZ 14153), *Aureobasidium pullulans* CF 10 (DSMZ 14940), *Aureobasidium pullulans* CF 40 (DSMZ 14941). Bio, CAN and HA belong to the BIOMIN Research Center culture collection.

Cell wall fragments were obtained by incubating the respective yeast strains in 1000 ml shaking flasks containing a 400 ml medium consisting of 1% soy peptone, 0.1% yeast extract, 0.2% magnesium sulfate heptahydrate, 0.2% ammonium sulfate, 1% saccharose and 2.5% glucose for 48 hours at 30°C and pH 5.0. The yeast cells were subsequently autolysed by incubating at pH 5 at a temperature of 50°C for 24 hours and centrifuged (17700 × g, 10 min) to separate cell wall fragments from the supernatant. Finally, the cell wall fragments were freeze dried (24 hours, -30°C/+30°C, 0.001 mbar) (Epsilon 2–90, Christ, Osterode, Germany). Autolysis was adapted according to Liu et al. (2008). The degree of autolysis was assessed by analysis of the supernatant fraction: cell rupture leads to increasing amounts of proteins and carbohydrates and dry matter in the supernatant. These parameters reached a maximum value after 24 hours. The progress of autolysis was also monitored by observing cells under the microscope (magnified 1000 times) and comparing them to cells from the original culture.

French press: Rupture took place in the French press with 1 cycle at 20,000 psi (=1,378.96 bar). Microscopic images are taken of the ruptured cells. Liquid supernatant was removed from the non-soluble cell walls by centrifugation (17700 × g, 10 min).

### Microplate-binding assay

#### ***Principle***

The quantitative adhesion of *E. coli* F4 (Bio 104 from BIOMIN Research Center culture collection) and *Salmonella* Typhimurium (Bio 99 from BIOMIN Research Center culture collection) on yeast products was determined by measuring the optical density of the culture solutions, which indicates the growth of the adhering bacteria. The growth rate depended on the number of adhering bacteria – with higher numbers bound resulting in faster growth and thus the bacteria entering earlier into the exponential phase.

#### ***Assay***

The yeast derivatives were suspended in phosphate buffered saline (PBS) in a concentration of 0.1 mg/L, the wells of a 96-well plate were coated with a 100 μL yeast suspension per well and incubated for 16–18 hours at 4°C. The plate was washed three times with PBS Tween20 using a microplate washer (Tecan 5082 M8/4R Columbus Plus). Subsequently, the bacterial suspension of *E. coli* F4 or *Salmonella* Typhimurium grown in Tryptic Soy Broth (TSB) overnight was adjusted to OD 0.01 and added to the wells of the microplate. Bacteria were allowed to adhere for 60 minutes at 37°C and the plate was subsequently washed 6 times with a microplate washer/PBS Tween20 to remove non-adherent bacteria. The wells were filled with 200 μL of TSB and overlaid with one drop of paraffin- oil. Blank, growth controls, negative control and binding control were also assessed.

The microplate was placed in a microplate reader (Tecan Genious), incubated for 18 hours at 37°C and OD was measured at a wavelength of 690 nm. Data were recorded every 15 minutes.

Serial dilutions of *E. coli* or *Salmonella* were prepared and applied in parallel on the microplate and the agar plate in order to establish a linear regression between the number of counted bacteria on the agar plate (CFU/mL) and the time in hours when the adhering bacterial solution reached the exponential phase (optical density of 0.1 at 690 nm). Independent calibration curves were created for the genera *E. coli* and *Salmonella*. Subsequently, the number of bacteria bound to the yeast product was determined using linear regression.

The binding control consisted of Bovine Serum Albumin (BSA) and test bacteria but without adding yeast to determine the non-specific binding sites. The binding control had no growth until after 6 hours. Finally the binding control, the bacterial number, which adhered to the non-specific binding sites of the well, was subtracted from the calculated data to determine the final number of specifically adhering bacteria. The blank consisted of the yeast cell wall product and BSA but without adding test bacteria and included all the assay steps to determine the bacterial number of the yeast cell wall product. The blank had no growth until after 10 h. The media control (TSB) had no growth until after 18 h.

The calibration is valid only for the respective genera and at an optical density of 0.1 (Ganner et al. 2010).

### Carbohydrate and protein analysis

For total mannan and glucan determination, yeast cell wall polysaccharides were hydrolyzed with 72% sulfuric acid. Carrez-precipitation was subsequently performed to remove interfering proteins and fats by precipitation as zinc (2+) and/or cyanoferrat (II) - complexes (Matissek et al. 1992). The cleared supernatants were analyzed for glucose and mannose by HPLC-RID using an ICSep ION-300 column (Transgenomic).

Total protein was determined by Total Kjeldahl Nitrogen (TKN, AOAC Official Method 954.01, 1954).

Carbohydrate analyses were conducted in single, protein analyses were conducted in duplicates.

### Statistical analysis

Experimental data were analyzed using IBM SPSS, version 19. Due to non-normal distribution of data, Spearman correlation was used. The calculated coefficient of correlation is only relevant for a given significance value (P-value <0.05); the coefficients are classified as below. A positive Spearman correlation coefficient corresponds to an increasing monotonic trend between *X* and *Y.* A negative Spearman correlation coefficient corresponds to a decreasing monotonic trend between *X* and *Y*. The influence of treatment (autolysis or French press) was calculated with the non-parametric Mann Whitney *U* Test (data non-normal distributed).

#### ***Coefficient of correlation classification***

Correlations were considered very low (r ≤ 0.2), low (0.2 < r ≤ 0.5), medium (0.5 < r ≤ 0.7), high (0.7 < r ≤ 0.9), very high (0.9 < r ≤ 1).

## Results

Different commercially available yeast cell wall products and autolysate products were tested for their ability to bind *E. coli* F4 and *Salmonella* Typhimurium: *E. coli* F 4 binding numbers ranged between 10^1^ and 10^3^ CFU/mg yeast; *Salmonella* Typhimurium binding numbers between 10^3^ and 10^4^ CFU/mg yeast. The analysis of the mannan-, glucan-, and protein content of the yeast products were also presented: Mannan contents ranged between 3% and 28%; glucan contents between 12% and 68%, and protein contents between 3% and 45% (Table 1).

**Table 1:** **Analysis of different yeast derivatives (3 replicates): amount of bound *E. coli *** **F4 and *Salmonella *** **Typhimurium (CFU/mg yeast); mannan-, glucan-, and protein-content (in % of dry matter)**

**Yeast derivative**	***E. coli *** **F4 (CFU/mg yeast)**	***Salmonella *** **Typhimurium (CFU/mg yeast)**	**Glucan (% DM)**	**Mannan (% DM)**	**Protein (% DM)**
cell wall 1	50, 50, 100	26000, 41000, 49200	37.75	19.71	26.40
cell wall 2	125, 115, 85	12200, 36900, 37900	28.81	11.97	30.7
cell wall 3	2300, 2100, 1900	80000, 153000, 125000	27.94	28.78	26.40
cell wall 4	-	1100, 300, 600	20.48	11.95	36.00
cell wall 5	35, 40	1300, 27000, 40000	26.94	6.92	30.90
autolysate 1	130, 100, 110	6000, 6500, 5400	12.60	5.14	45.80
autolysate 2	80, 230, 216	30000, 65000, 78000	23.62	14.75	40.10
autolysate 3	67, 95, 130	49000, 59000, 60000	22.84	16.39	38.20
autolysate 4	1200, 900, 1000	78000, 130000, 37000	23.08	17.68	38.10
autolysate 5	150, 350, 100	48000, 59000, 78000	22.66	16.15	36.40
Yeast derivative 1	216, 130, 100	8900, 6000, 9000	68.38	11.37	3.90
Yeast derivative 2	5, 10, 25	4000, 1000, 4000	59.04	3.25	5.30

The results of the Spearman correlation analysis revealed a statistically significant correlation between mannan content and *E. coli* F4 binding (P = 0.005) and between glucan content and *Salmonella* Typhimurium binding (P < 0.001). The coefficient of correlation was 0.315 (low correlation) for *E. coli* and mannan and 0.754 (high correlation) for *Salmonella* Typhimurium and glucan. No statistically significant correlation was found between glucan content and *E. coli* binding (P = 0.257) or between protein content and *E. coli* binding (P = 0.06). There was also no statistically significant correlation between mannan content and *Salmonella* binding (P = 0.07) or protein content and *Salmonella* binding (P = 0.21) (Table 2, Table 3).

**Table 2:** **Spearman correlation between quantity of bound *E. coli *** **F4 (CFU/mg yeast) and mannan-, glucan-, and protein-content of yeast derivatives**

			**Glucan [%]**	**Mannan [%]**	**Protein [%]**
Spearman-Rho	CFU E. coli [CFU/mg yeast]	Coefficient of correlation	-0.130	0.315	0.214
		Sig. (2-side)	0.257	0.005	0.060

**Table 3:** **Spearman correlation between quantity of bound *Salmonella *** **Typhimurium (CFU/mg yeast) and mannan-, glucan-, and protein-content of yeast derivatives**

			**Glucan [%]**	**Mannan [%]**	**Protein [%]**
Spearman-Rho	CFU Salmonella [CFU/mg yeast]	Coefficient of correlation	0.754	0.202	0.143
		Sig. (2-side)	0.000	0.077	0.210

Furthermore, we analyzed different yeast strains for their capability to bind *E. coli* F4 and *Salmonella* Typhimurium. *E. coli* binding ranged between 10^1^ and 10^3^ CFU/mg and *Salmonella* binding between 10^4^ and 10^5^ CFU/mg. Glucan contents ranged between 16% and 39%, mannan content between 3% and 23%, and protein contents between 14% and 39% (Table 4). No statistically significant correlation (Spearman correlation analysis) between the mannan-, glucan-, or the protein content and the binding numbers for *E. coli* and *Salmonella* Typhimurium were noted.

**Table 4:** **Analysis of different yeast strains (standardized laboratory fermentation and autolysis or French press treatment) on *E. coli *** **F4 and *Salmonella *** **Typhimurium binding capability (3 replicates); mannan-, glucan-, and protein-content**

**Yeast**	***E. coli *** **F4 (CFU/mg yeast)**	***Salmonella *** **Typhimurium (CFU/mg yeast)**	**Glucan (% DM)**	**Mannan (% DM)**	**Protein (% DM)**
*Saccharomyces cerevisiae* HA 282*	100, 900, 300	200000, 50000, 55000	19.09	23.67	39.41
Autolysis					
*Saccharomyces cerevisiae* HA 282*	1000, 400, 4000	600000, 170000, 80000	19.03	23.07	38.65
French press					
*Saccharomyces cerevisiae* HA 283*	3500, 2000, 1800	90000, 80000, 110000	16.81	23.84	35.08
Autolysis					
*Saccharomyces cerevisiae* HA 283*	100, 60, 10	260000, 100000, 120000	16.45	21.69	34.68
French press					
*Saccharomyces cerevisiae* CAN 214*	2000, 3000, 600	230000, 155000, 100000	15.09	20.41	36.12
Autolysis					
*Saccharomyces cerevisiae* CAN 214*	1700, 700, 270	350000, 90000, 45000	16.62	22.41	33.03
French press					
*Saccharomyces cerevisiae* Bio 19* (DSMZ 1848)	3000, 4000, 3500	160000, 100000, 165000	16.99	22.7	34.43
Autolysis					
*Saccharomyces cerevisiae* Bio 19* (DSMZ 1848)	100, 1500, 350	66000, 56000, 27000	16.41	20.92	33.94
French press					
*Klyveromyces marxianus* Bio 21* (DSMZ 5420)	2400, 1700, 2200	400000, 290000, 240000	28.34	4.1	43.63
Autolysis					
*Klyveromyces marxianus* Bio 21* (DSMZ 5420)	450, 4500, 450	150000, 100000, 86000	25.49	3.12	47.13
French press					
*Trichosporon mycotoxinivorans* Bio 328* (DSMZ 14153)	7000, 1700, 1500	35000, 30000, 25000	23.21	9.46	16.86
Autolysis					
*Trichosporon mycotoxinivorans* Bio 328* (DSMZ 14153)	400, 500, 2000	110000, 90000, 66000	24.69	9.66	14.53
French press					
*Aureobasidium pullulans* CF10* (DSMZ 14940)	3000, 3500, 1700	130000, 100000, 15000	39.37	4.95	35.51
French press					
Aureobasidium *pullulans* CF 40* (DSMZ 14941)	400, 2500, 1000	40000, 27000, 15000	31.90	5.33	32.94
French press					

*BIOMIN Research Center collection number.

The Mann Whitney *U* Test demonstrated an influence of the yeast autolysis on *E. coli* binding in contrast to French press treatment (P < 0.001). For *Salmonella* binding, no influence was noted due to autolysis or French press treatment (P = 0.27).

## Discussion

Yeast cell wall fractions and yeast autolysate products have been proposed to bind enteropathogenic bacteria and therefore to possess prophylactic properties in the gut for the control of selective pathogenic bacteria such as *E. coli* and *Salmonella.* There is much in the literature describing this adherence mechanism and mode(s) of action of yeast derivatives (Mourao et al. 2006; Oyofo et al. 1989; Sharon and Ofek 2000; Shoaf-Sweeney and Hutkins 2008; Snellings et al. 1997; Spring et al. 2000; Terre et al. 2007; Yang et al. 2008). However, no study has ever investigated the quantitative number of pathogens bound by different yeast derivatives. The influences of yeast strains and production treatments (autolysis or French press) have also not been investigated before in combination with pathogen binding. We consider this an important quality parameter for yeast cell wall products. Indeed, there was a lack of quantitative analytical methods to determine the total amount of bacteria bound by the yeast cell wall (Applegate et al. 2010). Yeast cell wall products are believed to act as anti-adhesive agents and are thus able to prevent intestinal bacterial attachment by providing alternative adhesion sites to enterobacteria which contain mannose-specific type I fimbriae such as *E. coli* and *Salmonella* spp. (Ofek et al. 1977). Shoaf-Sweeney and Hutkins (2008) defined the adhesion affinity by the valency of protein-oligosaccharide interactions. A single molecule would have a low affinity but the creation of individual protein subunits with numerous oligosaccharide binding sites assembled to a filamentous structure leads to increased target avidity. The present study demonstrates that mannan content is an important parameter for *E. coli* binding (r = 0.315, low Spearman correlation), and glucan content has a high correlation with *Salmonella* Typhimurium binding affinity (r = 0.754). According to the current results and further evaluating the available literature, we propose that there are many more unknown factors (apart from mannan and glucan content) influencing binding affinity of these pathogens.

In addition to the total amount of mannan and glucan, the three-dimensional structure of mannose chains within the yeast cell wall is another critical factor to consider. We observed that mannose chains have a strong influence on the binding behavior of different yeast cell wall products and are important for pathogen immobilization. This is supported by the observation that in contrast to yeast cell wall products (mannan content between 10-25%), a purified mannan standard (99%) showed no pathogen binding activity (Ganner et al. 2007, unpublished data). Indeed, quantitative, rather than qualitative assays are essential in order to evaluate the pathogen binding capacity of cell walls from different yeast sources and strains (Ganner et al. 2008). Ofek et al. (2003) explained that apart from adhesion-receptor interaction, hydrophobic and other non-specific interactions might be involved in the adhesion process.

Becker and Galetti (2008) investigated various food and feed components for alternative adhesion of intestinal *E. coli* and *Salmonella.* Yeast mannan-oligosaccharides, pumpkin, sesame seed extract, palm kernel extract and konjac gum were among the strongest binding matrices for enteropathogens. Adhesion matrices differ tremendously which gives rise to various interpretations and speculations on substantial binding factors while suggesting that unspecific and unknown binding properties are involved. Moreover, taking into account the diversity of natural products and biological variation therein, the binding capacity of fibrous food and feed components for different bacteria seems rather unpredictable (Becker and Galetti, 2008).

Our observation that mannan content is strain dependent (Table 4) agrees with other studies (Jaehrig et al. 2008; Klis et al. 2002; Molnar et al. 2004). However, in our experiment with different yeast strains fermented under standardized laboratory conditions, we could not obtain any statistically significant correlations between mannan or glucan content and pathogen binding. According to these results, the yeast strain itself has no influence on *E. coli* F4 or *Salmonella* Typhimurium binding. However, we could demonstrate that the downstream process autolysis has an influence on *E. coli* binding (P < 0.001). Giovani et al. (2010) studied the effect of yeast strain and fermentation conditions on the release of cell wall polysaccharides. Temperature, media and sugar composition influenced the release of cell wall polysaccharides significantly. These data underline and support our results on the relative influence of production processes and production conditions on binding capacity.

The binding mechanism between pathogens and the yeast cell wall can be speculated, as demonstrated by the autolysis production process, the mannan content and the glucan content. However, as these binding mechanisms are only partially understood, we propose that other factors such as the cell wall structure, strain diversity, structural surroundings-, and non-specific interactions might also play important roles in pathogen immobilization, in addition to the total amount of mannan or glucan.

Summarizing, the present study demonstrates a variation among different yeast products concerning their capability to bind *E. coli* F4 and *Salmonella* Typhimurium. *Salmonella* Typhimurium was bound one to two orders of magnitude more than *E. coli* F4. We could demonstrate a statistically significant correlation between mannan and glucan content and pathogen binding. Furthermore we could demonstrate a relative influence of the production process autolysis.

However, the binding mechanisms involved are still not fully understood and our results underline the complexity of the present subject. The recently suggested mannose specific type-I fimbriae play a role and furthermore, unknown mechanisms which might interact synergistically are probably involved. Biological variation and natural diversity make predictions even more difficult and comprehensive. Further research is necessary, e.g., on a molecular level and a detailed characterization of cell wall components and cell wall structure, to elucidate on the additional forces which might be involved in the binding mechanism between yeast derivatives and enteropathogenic bacteria. Finally, additional standardized *in vivo* experiments with challenged animals could also further validate the binding potential of yeast products and the study of the anti-adherence strategy.

## Competing interests

The authors declare that they have no competing interests.
